# Does compulsory schooling affect health? Evidence from ambulatory claims data

**DOI:** 10.1007/s10198-021-01404-y

**Published:** 2021-11-15

**Authors:** Tatjana Begerow, Hendrik Jürges

**Affiliations:** Schumpeter School of Business and Economics, University of Wuppertal, Rainer-Gruenter-Str. 21 (FN), 42119 Wuppertal, Germany

**Keywords:** Compulsory schooling laws, Doctor-diagnosed health conditions, Regression discontinuity design, Causality, I14, I24, I26

## Abstract

Using claims data on more than 23 million statutorily insured, we investigate the causal effect of schooling on health in the largest and most comprehensive analysis for Germany to date. In a regression discontinuity approach, we exploit changes in compulsory schooling in West Germany to estimate the reduced form effect of the reforms on health, measured by doctor diagnoses in ICD-10 format covering physical as well as mental health conditions. To mitigate the problem that empirical results depend on subjective decisions made by the researcher, we perform specification curve analyses to assess the robustness of findings across various model specifications. We find that the reforms have, at best, very small impacts on the examined doctor diagnoses. In most of the specifications we estimate insignificant effects that are close to zero and often of the “wrong” sign. Therefore, our study questions the presence of the large positive effects of education on health that are found in the previous literature.

**Supplementary Information:**

The online version contains supplementary material available at 10.1007/s10198-021-01404-y.

## Introduction

This paper aims at contributing to the growing literature on the causal effect of education on health. Although there is consistent evidence that more educated people are healthier and live longer [1], it is still unclear to what extent the education-health relationship is causal. The question whether it is causal is, however, particularly relevant for the formation of education and health policies. Provided that there is a causal effect of education on health, education policies might be a cost-effective tool to improve population health, which is certainly one of the highest priorities for policy makers, especially against the background of the demographic change and the huge healthcare costs involved. Such policy interventions aimed at promoting health through educational initiatives are regularly proposed by international organisations such as the World Health Organization (WHO) and Organisation for Economic Cooperation and Development (OECD) [2, 3].

In the theoretical literature, several mechanisms have been suggested to explain the link between education and health. One often-discussed channel is productive efficiency, that is, education enters as a factor in the health production function and raises the efficiency in health production. Thus, more educated people are able to produce better health outputs from given quantities of health inputs [4]. For example, they are able to understand and follow the doctor’s instructions, which raises the benefits of doctor visits and results in a more effective treatment. Furthermore, education can improve health through a better choice of health inputs by improving the knowledge on the relationship between health behaviours and outcomes, referred to as allocative efficiency [5]. Put differently, more educated people are better at obtaining, evaluating and processing health information and may therefore be able to improve their health by using a more efficient mix of health inputs. For instance, more educated individuals are likely to be better informed about the adverse effects of smoking and consequently they are more likely to smoke less or to quit smoking. Finally, in addition to the two direct pathways, education may affect health indirectly through other channels. For example, better education may increase earnings [6] allowing people to buy healthier food, to purchase a gym membership or to pay higher housing prices to live in healthier regions with less traffic and pollution.

Despite the theoretical pathways discussed above, the interpretation of the association as causal is difficult because education is most likely endogenous. First, the correlation may come from unobserved confounder variables that affect both educational attainment and health such as parental characteristics, cognitive ability or time preferences [7]. Second, the relationship may be driven by reverse causality since poor health in childhood can lead to lower educational attainment [8]. If unobserved confounders and reversed causality are not controlled for, the estimate of the causal effect of education on health is likely to be biased. Following Angrist and Krueger [9], a growing number of studies have exploited exogenous variation in education caused by changes in compulsory schooling laws using instrumental variables (IV) or regression discontinuity (RD) techniques to estimate causal effects of education on various outcomes, including health and mortality.

Lleras-Muney [10] was one of the first to estimate the causal impact of education on mortality exploiting compulsory schooling laws and found very large effects on the probability of dying in the next 10 years in the United States^1^. Much smaller effects of education on mortality have been found by van Kippersluis et al. [12] for the Netherlands, by Fischer et al. [13] for Sweden and by Davies et al. [14] for the UK. Gathmann et al. [15] evaluate several compulsory schooling reforms implemented in Europe during the twentieth century and find small negative effects on mortality for men, but not for women. Furthermore, it appears that reforms implemented in the early twentieth century have a stronger impact on mortality than later reforms. Recently, Grytten et al. [16] find evidence, for Norway, suggesting that education has strong causal effect on mortality for men—to a large part due to fewer accidental deaths. Other studies, however, find no evidence for a causal effect of education on mortality, e.g. Albouy and Lequien [17] for France, Lager and Torssander [18] and Meghir et al. [19] for Sweden, Clark and Royer [20] for the UK and Mazumder [11] and Fletcher [21] for the United States. The latter findings are in line with those of a recent study by Malamud et al. [22], for Romania, suggesting no mortality reductions due to additional education.

Another set of studies estimates causal effects of education on self-rated health and health-behaviours and their findings also offer contradictory evidence. Mazumder [11], Fletcher [21], Silles [23] and Li and Powdthavee [24] find evidence for the US, the UK and Australia, showing that education improves self-rated general health. These findings are consistent with those by Brunello et al. [25] in several European countries in a multi-country framework. Moreover, Kemptner et al. [26] find evidence for a protective effect on self-reported health for men but not for women in West Germany. Some studies suggest that education also improves mental health [27, 28]. In addition, Kemptner et al. [26], Brunello et al. [29], James [30] and Dursun et al. [31] report that schooling has a negative effect on obesity and BMI, while Silles [32] finds that additional education lowers the probability of smoking for males. In contrast, other studies provide no support for a causal effect of education on self-reported general health [20, 30, 31, 33–35], BMI and other weight-related outcomes [24, 33, 34], mental health [36, 37] or smoking behaviour [20, 24, 26, 30, 31, 33, 34, 38]. Recent evidence on the causal effect of education on self-reported health is provided by Fonseca et al. [39], Janke et al. [40], Albarrán et al. [41], Dilmaghani [38] and Malamud et al. [22]. Fonseca et al. find that education has a positive effect on a wide range of self-reported health measures including functional status, instrumental functional status and chronic conditions when evaluating several reforms in the US, the UK and Continental Europe. In contrast, Janke et al., analysing two UK education policy reforms, report that education has no effect on a number of self-rated chronic conditions, except for diabetes. Albarrán et al., Dilmaghani and Malamud et al. find no evidence that education has an effect on self-rated health.

The literature on the causal effect of education on more objective measures of health such as biomarkers of health is relatively scarce, which was also investigated by Hamad et al. [42] in a recent review and meta-analysis. Indeed, only four studies so far have focused on biomarkers of health. In addition, these studies have also generated different results. Powdthavee [43] exploits two compulsory schooling laws in the UK in 1947 and 1973 to examine the causal effect of education on hypertension as important predictor of heart disease and finds a reduction in hypertension for the first law in 1947, but no effect for the second law in 1973. Exploiting the same reforms, Jürges et al. [44] and Clark and Royer [20] report no effects on inflammatory blood biomarkers and on blood pressure, respectively. More recently, Courtin et al. [45] evaluate the 1959 Berthoin compulsory schooling law in France and find no evidence that education has an effect on 16 biomarkers of cardiovascular, immune, metabolic and organ function.

Overall, the literature does not come to a consensus to what extent the link between education and health and longevity is causal. This has also been investigated by Hamad et al. [42], showing that the findings from quasi-experimental studies using compulsory schooling reforms to estimate causal effects of education on health are mixed with some studies finding protective effects and others reporting no effects. Hence, the debate on whether the correlation between education and health can be interpreted causally is still ongoing, as a considerable amount of recently published studies have shown [16, 22, 33, 36–41, 45, 46]. Lately, Xue et al. [46] performed a meta-analysis on the literature on the causal effect of education on health. Their findings show that the exiting literature suffers from publication bias in favour of positive results and that the effect of education on health is close to zero after correcting for this publication bias.

We contribute to the existing literature in four important ways. First, we perform the largest and most comprehensive analysis on the causal effect of education on health for Germany to date. Our data set contains ambulatory care insurance claims of overall 23.6 million statutorily insured and thus almost 90% of the German population in the relevant cohorts [47]. To the best of our knowledge, this is the largest data set that has ever been used to study the causal effect of education on health. Second, our data set allows us to study the causal effect of education on a wide range of doctor-diagnosed diseases coded according to the International Classification of Diseases 10 (ICD-10) that cover physical as well as mental health conditions. So far researchers have mostly used self-reported health measures. However, it is often discussed that these measures are subject to reporting bias as people may under-, over-, or misreport their health for certain reasons (e.g. understanding of questions, financial incentives). This is problematic because these factors tend to vary with socioeconomic characteristics such as education. Therefore, objective health measures are often preferred over self-reported measures [48]. Since our data set contains doctor-diagnosed conditions we are able to study the causal effect of education on health measures that are “more objective” compared to self-reported health measures. Third, our data set allows us to use month of birth instead of year or quarter of birth to assign the treatment status, which leads to more precise estimates.^2^ Finally, we perform specification curve analyses to assess the robustness of findings for all reasonable model specifications to account for subjective analytic decisions by the researcher that might affect empirical results [50].

Following Pischke and von Wachter [51] and Kemptner et al. [26], we exploit reforms in West Germany implemented between 1946 and 1969 that raised compulsory schooling from 8 to 9 years in the basic track of secondary schooling. We identify the reduced form effect of the compulsory schooling reforms on doctor-diagnosed conditions in an RD approach. Our results indicate that the reforms have, at best, very small impacts on doctor diagnoses. In most of the specifications we estimate insignificant effects that are close to zero and often of the “wrong” sign. Therefore, our study questions the presence of the sometimes quite large positive effects of education on health that are found in the previous literature and casts doubt on the effectiveness of policies aimed at promoting population health through educational interventions.

The remainder of the paper is organized as follows. We first describe the empirical strategy and the data. We then present the results and their robustness to several different specifications. Finally, we discuss our findings and conclude.

## Empirical strategy

### Compulsory schooling reforms in Germany

To deal with the potential endogeneity of education, we exploit reforms in West Germany that increased compulsory basic track schooling from 8 to 9 years.^3^ For a detailed description of the German school system see Online Appendix A.

According to Petzold [58] the introduction of the reforms had three main objectives. First, there were pedagogical and psychological arguments, mainly presented by pedagogues, psychologist and physicians, who argued that students were too young and immature for the labour market after 8 years of schooling. Sending 14-year old students at the beginning of puberty to the labour market was further considered to be dangerous for their mental and physical development. The additional ninth grade also aimed at preparing students for their working life. The idea was to provide students guidance in choosing an occupation in the additional school year, since they were considered not mature enough to choose an occupation after 8 years in school. Second, political parties, associations and institutions expected a mitigation of youth unemployment and the social changes involved. The period after World War II was characterized by a high level of youth unemployment and a shortage of vacant apprenticeships. Therefore, the additional school year was sometimes seen as an “institutional storage” of students, who would have been unemployed otherwise. Third, arguments with respect to educational economics became prevalent at the end of the 1950s. Due to technical progress it became necessary to have more intellectual instead of manual workers. The additional school year therefore aimed at guiding youths away from manual to more intellectually demanding jobs by providing longer and more academic education.

Since in Germany the federal states are responsible for education policy, the ninth year of compulsory schooling was introduced at different points in time in the federal states. Table 1 reports by federal state the year and month of implementation and the first month-year of birth cohort of individuals affected by the reforms. While the two northern states Hamburg and Schleswig-Holstein already introduced the reform in the 1940s, the other states followed in the late 1950s and 1960s. In Saarland the compulsory ninth grade was implemented in 1958, Bremen decided to introduce it in 1959 after having several years of a voluntary ninth grade. In Lower-Saxony the additional school year was already determined by law in 1954, but the overall introduction was in April 1962. The remaining states introduced the compulsory ninth grade due to the so-called Hamburg Accord (“Hamburger Abkommen”) in 1964, in which the prime ministers of the federal states agreed on the introduction by 1967 at the latest [51]. As a consequence, North Rhine-Westphalia introduced it in 1966, followed by Baden-Wuerttemberg and Rhineland-Palatinate in 1967. In Hesse some schools that already fulfilled the organisational requirements started to introduce it in 1962, but the overall implementation followed in 1966. Bavaria was the last state that implemented the ninth grade in 1969.^4^

**Table 1:** Introduction of the ninth year of compulsory schooling in the basic track

Federal state	Reform implementation	First birth cohort affected
Hamburg	April 1946	April 1931
Schleswig-Holstein	April 1947	April 1932
Saarland	April 1958	April 1943
Bremen	April 1959	April 1944
Lower Saxony	April 1962	April 1947
Hesse	April 1966	April 1951
North Rhine-Westphalia	April 1966	April 1951
Rhineland-Palatinate	April 1967	April 1952
Baden-Wuerttemberg	April 1967	April 1952
Bavaria	August 1969	August 1954

Notes: The table reports by federal state the year and month of implementation and the first month-year of birth cohort of individuals affected by the compulsory schooling reforms. The pivotal birth cohort is calculated assuming that children enter school with the beginning of the school year after turning 6 years old

In some federal states the compulsory schooling reform coincided with another reform that shifted the start of the school year from April to August in all federal states except for Bavaria, which started the school year in August already. This transition had to be performed by the beginning of the school year 1967. As a consequence, eight of the ten states decided to have two short school years of 8 months each. Hamburg implemented a long school year [60]. Baden-Wuerttemberg, Hesse, North Rhine-Westphalia and Rhineland-Palatinate were affected by these simultaneous implementations since they introduced the additional year of compulsory schooling with the beginning of the school year 1966. A potential problem with the introduction of the short school years is that the first cohorts after the introduction of compulsory schooling reform formally completed 9 instead of 8 school years although they did not spent much more time in school compared to the previous cohorts who were subject to 8 years of compulsory schooling. Thus, the true amount of schooling of the affected cohorts is over-estimated. However, Kemptner et al. [26] address this issue and show that their estimates are robust to recoding the education variable, indicating that the short school years should not present a major problem for our analysis.

### Regression discontinuity design

**Fig. 1:**
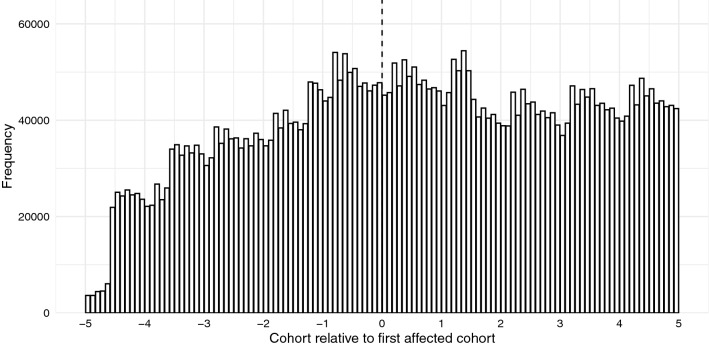
The figure shows a histogram of the assignment variable (month-year of birth) for five cohorts before and after the first birth cohort affected by the reforms. There is no evidence for bunching at the cut-off and therefore no evidence of manipulation of the assignment variable. Source: KVB claims data

To estimate the reduced form effect of compulsory schooling on health we use an RD design.^5^ The key identifying assumption for an RD design to be valid is that all observable and unobservable factors are continuously related to the assignment variable, that is, there are no discrete jumps at the cut-off. In case of unobservable factors, this assumption cannot be tested, but it is common practice to provide evidence on its plausibility by showing that no observable covariates discontinuously change around the cut-off (see Lee and Lemieux [62]). However, this is not possible in our case since the claims data only contain information on year and month of birth, federal state, gender and doctor diagnoses, but not on other potential covariates. In addition, the continuity assumption may not be plausible if individuals are able to manipulate their treatment status to be on one particular side of the cut-off. It is reasonable to assume that parents were unable to manipulate their children’s month-year of birth because the birth date is determined considerably before the announcement of the policy. To assess the potential manipulation of the assignment variable we show a histogram of the assignment variable for observations above and below the cut-off. The visual inspection in Fig. 1 shows that there is no sign of manipulation of the assignment variable. The distribution of month-year of birth shows some random jumps, but apparently no jump at the cut-off.

The regression model to estimate the reduced form effect of the compulsory schooling reforms on health, is specified as follows:

1 $$\begin{aligned} H_c = \alpha + \beta D_c + f(R_c) + \gamma X_c + \epsilon _c, \end{aligned}$$

$H_c$ denotes the health status of individuals in month-year of birth cohort *c*. $D_c$ is an indicator variable, which takes the value 1 if month-year of birth cohort *c* is affected by the reform and therefore required to stay 9 years in school and 0 otherwise. $R_c$ measures the birth cohort relative to the relevant cut-off and is positive for cohorts who are affected by the reform and negative for cohorts who are not affected by it. In the basic specification, $X_c$ includes year of birth, month of birth and federal state fixed effects to account for differences across birth years, months and federal states. Moreover, we include a quadratic age term to address potential non-linear age effects. $\epsilon _c$ denotes the error term. Since the data are aggregated we run regressions that are weighted by the number of individuals in a cell. Moreover, the equation is estimated separately for men and women and with standard errors clustered by federal state $\times$ month-year of birth. The reform effect of interest is $\beta$.

The function $f(\cdot )$ captures the relationship between the assignment variable and the outcome and needs to be correctly specified. Since a misspecification of the functional form can generate biased estimates of the treatment effect, it is recommended by Lee and Lemieux [62] not to rely on one specification. More precisely, it should be demonstrated that the estimates are not sensitive to the chosen bandwidth by presenting estimates for various bandwidths. Furthermore, it is advisable to report estimates for various trend specifications. We follow these recommendations and show results of local linear and quadratic as well as global linear, quadratic, cubic and quartic regressions based on samples with bandwidths of 1, 3, 5, 7 and 9 cohorts around the cut-off. The results are presented graphically in specification curves.

### Specification curve analysis

When specifying a causal model researchers have various options. For instance, they can decide on the control variables to use, the observations to exclude and the functional form to assume. In economics it is therefore standard practice to test a few different model specifications to demonstrate the robustness of results. Regression results are usually shown in tables with multiple columns capturing different specifications, which can be very complex and confusing when lots of specifications are reported. This might be problematic because the reported results depend on an arbitrary choice made by the researcher. Probably even more problematic is that researchers often have a conflict of interest and selectively report evidence from specifications that yield the desired results. Undesirable or unexpected results are often not reported, which is referred to as reporting bias. Consequently, it is not surprising that analyses on the same research question and data often lead to contradictory results. Building on that, a so-called specification curve analysis offers a possibility to provide a more comprehensive analysis by assessing the robustness of findings for all “reasonable” model specifications, i.e. all specifications that the researcher considers as non-redundant and valid. The basic idea behind a specification curve analysis is to define a set of reasonable specifications, to estimate all of them and to report the results graphically in a descriptive specification curve that displays the range of estimates that are obtained through alternative specifications. Hence, specification curve analysis reduces the problem of selective reporting by researchers [50].

To answer the question whether education affects health in an RD approach, we identified three major analytic decisions: first, the functional form of the relationship between the outcome and treatment-determining variable to assume (non-parametric/local versus parametric/global estimation), second, the bandwidth around the cut-off to use, and third, the covariates to include. Regarding the functional form we found it reasonable to estimate local linear and local quadratic regressions as well as global regressions including first-order to fourth-order polynomials in birth cohort. With respect to the bandwidth choice we considered it as sufficient to run models on samples of five different bandwidths.^6^ The third analytic decision is related to the inclusion of covariates. Our basic model includes year of birth, month of birth and federal state fixed effects as well as a quadratic age term. However, it is also reasonable to assume that some federal states have flatter or steeper increases in average educational attainment or health than others. Hence, it might be useful to include also state-specific trends to control for state-specific deviations from the nationwide trend captured by the cohort fixed effects. Accordingly, we decided to run models controlling for linear, quadratic and cubic state-specific cohort trends in addition to the control variables mentioned above as well as models without controlling for state-specific trends at all. Moreover, we ran separate analyses for men and women since health benefits of education may differ by gender. The combination of the analytic decisions mentioned above resulted in 240 different specifications to estimate for each considered outcome variable (6 functional forms $\cdot$ 5 bandwidths $\cdot$ 4 state-specific trends $\cdot$ 2 sexes).

## Data

Our analysis is based on data that has been provided by the National Association of Statutory Health Insurance Physicians (Kassenärztliche Bundesvereinigung, KBV)^7^. The data set includes all statutorily insured German inhabitants born between January 1930 and December 1959 with at least one ambulatory care claim recorded in 2009 (*N* = 23.6 million), which is equivalent to about 90% of the overall German population in the relevant cohorts in 2009 [47]. Individual claims contain information on primary and secondary diagnoses in ICD-10 format and are aggregated into grouped data containing information on the number of individuals having a condition on record and the total number of individuals. Based on that, we calculated the proportion of patients who generated a claim to the statutory health insurance related to a specific ICD-10 code, which we refer to as morbidity rates.

Table 2 shows the nine health conditions with the corresponding ICD-10 codes that were selected for the analysis. The selection of the diseases was largely guided by the literature. Since the risk of chronic diseases has been found to be higher for people with lower socioeconomic status [66], we included ischemic heart disease (ICD I20-I25), diabetes mellitus (ICD E10-E15) and chronic lower respiratory diseases (COPD, ICD J40-J47) as well as obesity (ICD E66) as major risk factor for these chronic diseases. It is also known that a higher socioeconomic status is associated with a lower risk for various types of cancer (ICD C00-C97) [70]. Musculoskeletal diseases (ICD M00-M99) in general and back pain (ICD M54) in particular are also interesting to study since they are negatively related to the socioeconomic status [71]. The negative link can be explained by occupational characteristics as better educated people tend to work in non-manual occupations with safer and healthier working conditions, which may reduce the risk of musculoskeletal diseases, including back pain. Furthermore, the probability to develop a depression (ICD F30-F39) is known to be lower for people with a higher socioeconomic status, most probably because it increases the access to higher occupations involving factors such as direction, control, and planning, which may protect against depression [72]. Finally, we selected urogenital diseases (ICD N00-N99) because they have not been studied so far in the education-health literature. However, urogenital diseases are interesting to look at because some types may be the result of unhygienic conditions and practices that are probably more common in lower socioeconomic groups [66].

**Table 2:** Selection of diagnoses with corresponding ICD-10 codes

ICD-10 code	Diagnosis
I20-I25	Ischemic heart disease
E10-E15	Diabetes mellitus
E66	Obesity
F30-F39	Depression
J40-J47	Chronic lower respiratory diseases (COPD)
C00-C97	Malignant neoplasms (cancer)
M00-M99	Diseases of the musculoskeletal system (musculoskeletal diseases)
M54	Back pain
N00-N99	Diseases of the genitourinary system (urogenital diseases)

Notes: The table shows the selection of diseases with the corresponding ICD-10 codes. The selection of diseases for the analysis was largely guided by the literature

Ideally, we would need information on the federal state of last school attendance to ensure a precise assignment of the treatment. However, the claims data only contain the federal state of residence in 2009. Using this as a proxy, we implicitly assume that individuals attended school in the federal state where they lived in 2009 and thus, the treatment might be imprecisely assigned if cross-state mobility in Germany exists. This measurement error in the treatment indicator would then downward bias our estimates of the reduced form effect. However, in additional analyses using SOEP data, we show, that cross-state mobility in Germany is quite low. Moreover, we find that reduced form point estimates for the effect of the compulsory schooling reforms on self-reported health status are almost identical when using the federal state of residence compared to the federal state of last school attendance (see Online Appendix B). Therefore, we are confident that it is not a major problem to proxy the state of school attendance with the state of residence.

We restrict our sample to German inhabitants living in the ten West German federal states, excluding Berlin. The reason for excluding former East Germany is that, to the best of our knowledge, there was no comparable compulsory schooling reform. Berlin is excluded because it is difficult to identify whether an individual attended school in West or East Berlin. For the analysis we only consider individuals with valid information on year of birth, month of birth, federal state of residence and doctor diagnoses. For our baseline specification, we select individuals born 5 years before or after the first affected cohort of each compulsory school reform (*N* = 6,202,659).

Descriptive statistics in Table 3 show that the final sample consists of little less men than women. On average, individuals are born in 1951 and are 58 years old. In the sample, the morbidity rate for musculoskeletal diseases is highest (67.6%), followed by urogenital diseases (44.0%). The lowest morbidity rates are found for ischemic heart disease (12.5%) and cancer (10.1%)^8^. The morbidity rates for the selected diagnoses are in the same range for men and women, but there are three exceptions. First, ischemic heart diseases are more than twice as prevalent among men (17.1% versus 7.9%). This finding is generally in line with the literature, reporting that men develop ischemic heart disease earlier in life than women, although they share many cardiovascular risk factors. One important reason for the lower age-specific risk in women is that estrogen has a positive effect on the cardiovascular system that can protect women from ischemic heart disease before the menopause [67].

**Table 3:** Descriptive statistics

	(1)	(2)	(3)
	Full sample	Men	Women
Demographics			
Age	58.4 (5.1)	58.4 (5.0)	58.4 (5.1)
Year of birth	1950.6 (5.1)	1950.6 (5.0)	1950.6 (5.1)
Male (%)	45.0		
Health status			
Ischemic heart disease (%)	12.5	17.1	7.9
Diabetes mellitus (%)	18.1	21.5	14.8
Obesity (%)	14.1	12.6	15.6
Depression (%)	17.5	12.1	22.8
COPD (%)	20.5	20.8	20.1
Cancer (%)	10.1	10.4	9.8
Musculoskeletal diseases (%)	67.6	64.6	70.6
Back pain (%)	36.5	33.9	39.0
Urogenital diseases (%)	44.0	31.5	56.4
*N*	6,202,659	2,788,562	3,414,097

Notes: The table reports mean values of all variables as well as standard deviations of all continuous variables in parentheses for the pooled sample of men and women (column 1) and for the sub-sample of men (column 2) and women (column 3). Source: KBV claims data

Second, Table 3 shows that depression is about twice as prevalent among women (22.8% versus 12.1%). This is also consistent with the literature, suggesting that depression is more common in women, which has typically been explained by hormonal changes during puberty, pregnancy, postpartum or during the transition to menopause. Moreover, socially-driven risk factors may contribute to the increased prevalence of depression in women [68]. Third, urogenital diseases are roughly 60% more likely among women (56.4% versus 31.5%), as shown in Table 3. This is also in line with the literature on risk factors for genitourinary diseases in general and urinary tract infections in particular, reporting that urinary tract infections are more common among women. Typical risk factors explaining the increased prevalence in women are anatomic factors as well as sexual intercourse and the use of spermicidal contraceptives [69].

## Results

**Fig. 2:**
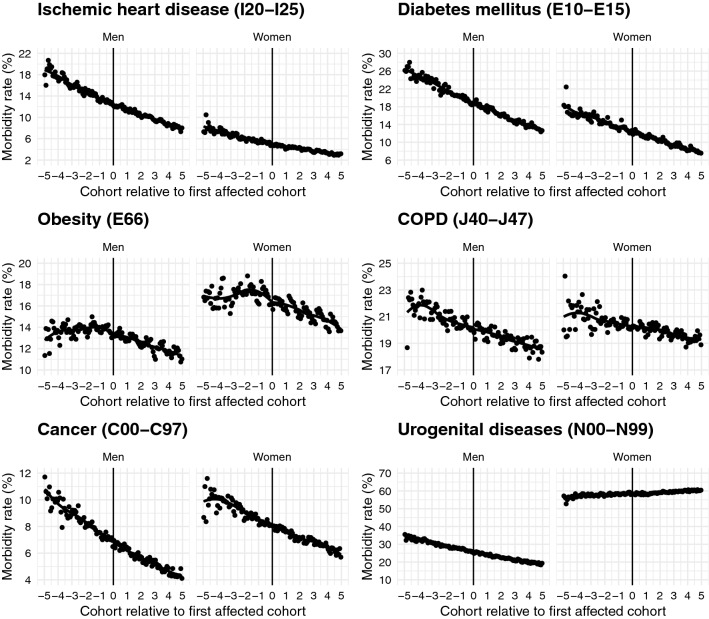
The figure plots morbidity rates in percent by month-year of birth for five cohorts before and after the first birth cohort affected separately for men and women. All data points in the figure present averages by month-year of birth. The vertical line denotes the first birth cohort affected by the law changes. Source: KVB claims data

Figure 2 plots morbidity rates in percent by month-year of birth for five cohorts before and after the first birth cohort affected separately for men and women and gives a first graphical impression on whether the reforms had an effect on doctor-diagnosed health conditions. The figure shows morbidity rates that decrease in month-year of birth (i.e. increase in age) but do not change discretely at the cut-off. Apart from different prevalences, we find no considerable differences between men and women.

It is important to mention that depression, musculoskeletal diseases and back pain are not part of the figure. The reason is that we found an increase in the probability to be diagnosed with these conditions before 1950 and a sudden decrease after 1950 (see Online Appendix C), which represents individuals at age 59 who gradually start to retire. Therefore, the observed trend possibly reflects a “retirement effect” as musculoskeletal diseases including back pain and depression are more likely to be diagnosed and treated when individuals are still in the labour market because a medical certificate is needed to take sickness leave. Retired individuals do not need this anymore. Moreover, back pain and depression are among the most important drivers of early retirement in Germany [73]. As the sudden decrease in the percentage treated after age 59 is not random and therefore threatens our identification strategy, we decided to exclude these conditions from further analyses. This resulted in the six remaining doctor diagnoses presented in Fig. 2.

**Fig. 3:**
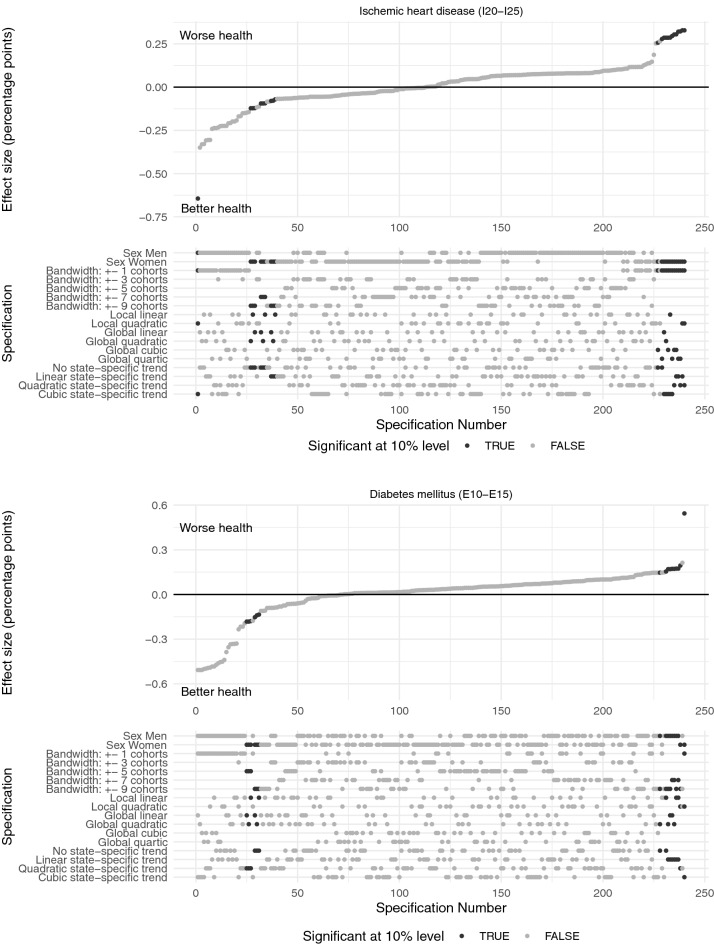
The figure shows the results of the specification curve analyses of the effect of the reforms on the probability to suffer from ischemic heart disease (I20-I25) and diabetes mellitus (E10-E15), respectively. Each dot in the top panel represents a point estimate from a different model specification. The specifications are ordered by effect size. The dots vertically aligned below indicate the combination of analytic decisions behind those estimates. Dark grey dots represent significant effects ($p < 0.1$), while light grey dots show insignificant effects. Source: KBV claims data

**Fig. 4:**
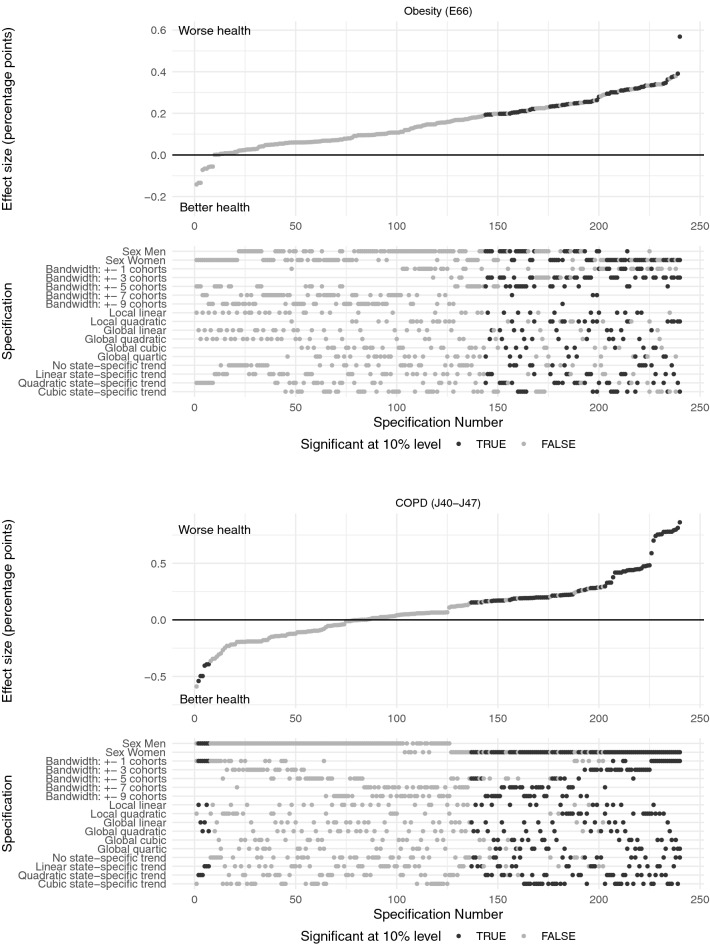
The figure shows the results of the specification curve analyses of the effect of the reforms on the probability to suffer from obesity (E66) and COPD (J40-J47), respectively. Each dot in the top panel represents a point estimate from a different model specification. The specifications are ordered by effect size. The dots vertically aligned below indicate the combination of analytic decisions behind those estimates. Dark grey dots represent significant effects ($p < 0.1$), while light grey dots show insignificant effects. Source: KBV claims data

**Fig. 5:**
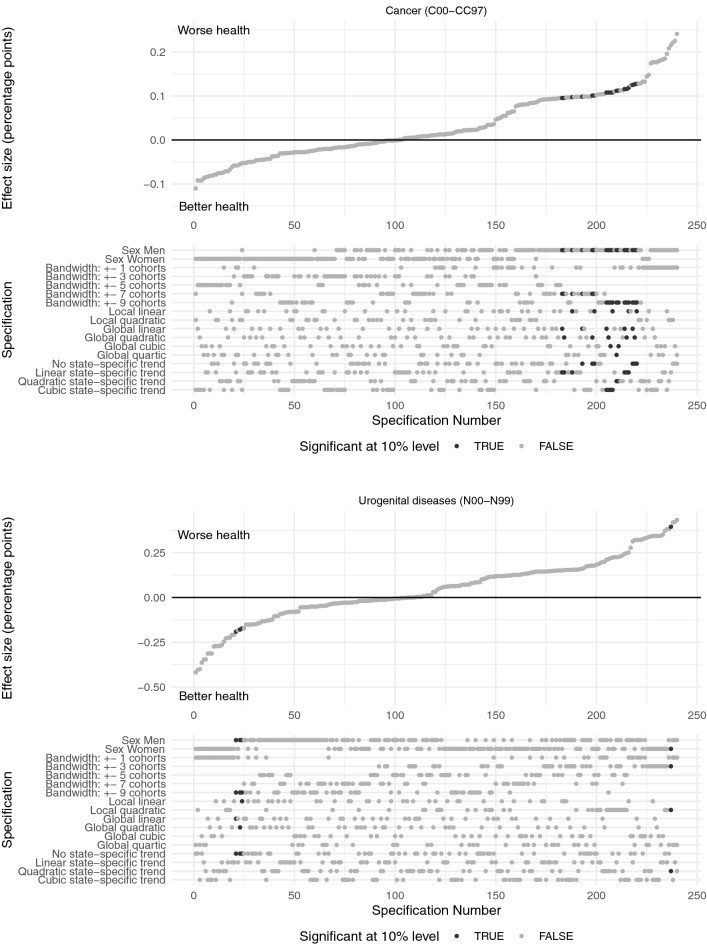
The figure shows the results of the specification curve analyses of the effect of the reforms on the probability to suffer from cancer (C00-C97) and urogenital diseases (N00-N99), respectively. Each dot in the top panel represents a point estimate from a different model specification. The specifications are ordered by effect size. The dots vertically aligned below indicate the combination of analytic decisions behind those estimates. Dark grey dots represent significant effects ($p < 0.1$), while light grey dots show insignificant effects. Source: KBV claims data

As described above, we performed specification curve analyses to report the reduced form regression results of the effect of the compulsory schooling reforms on doctor diagnoses graphically. Specification curves for ischemic heart disease and diabetes mellitus are presented in Fig. 3, for obesity and COPD in Fig. 4 and for cancer and urogenital diseases in Fig. 5. Each dot in the top part of each specification curve represents a point estimate from a different model specification. The 240 different specifications are ordered by effect size. The dots vertically aligned below indicate the combination of analytic decisions behind those estimates. Dark grey dots represent effects that are significant at the 10% level, while dots in light grey show insignificant effects.

The specification curves show that there is only very little empirical support for an effect of the West German compulsory schooling reforms on health and thus confirm the graphical impression in Fig. 2. All six specification curves show very similar results with coefficients that are centred around zero. For example, for cancer the effect sizes range from $-$ 0.1 to 0.2% points. Given the baseline probability of suffering from cancer in the sample of about 10% these estimates reflect basically zero effects. For COPD effect sizes are somewhat larger ($-$ 0.6–0.9% points on a basis of 20%), but still, they are very small.

In the presence of a morbidity-reducing effect of the compulsory schooling reforms, we would expect the estimated coefficients to have a negative sign throughout all specifications. Surprisingly, we find more positive than negative coefficients for all the considered health conditions indicating that the compulsory schooling reforms actually *increased* the probability to suffer from these diseases. One extreme case is obesity with 96% positive coefficients that are, however, also close to zero.

Moreover, across all six specification curves the estimated effects are mostly insignificant with only 2–10% of coefficients that are significant at the 10% level. One exception is COPD (J40-J47) with significant coefficients in 40% of the considered specifications. However, some of the significant effects on the probability of being diagnosed with COPD are negative and some positive. We find, that the compulsory schooling reforms significantly decrease the probability to suffer from COPD for men and significantly increase it for women. Coefficients for the probability to suffer from ischemic heart disease (I20-I25) that are significant at the 10% level are almost exclusively found in the female sub-sample. Inconsistently, some of the significant effects among women are negative and some positive. For the probability to be diagnosed with cancer (C00-C97) we only find significant positive coefficients pointing to an *increase* of the probability to suffer from cancer due to the reforms. These effects are solely observed in the male sub-sample. Moreover, we find some support for a significant *increase* in the probability to suffer from obesity (E66) for both sexes. For diabetes mellitus (E10-E15) we find significant negative effects among women and significant positive effects among men. The effect of the compulsory schooling reforms on urogenital diseases (N00-N99) is only significant in 2% of specifications.

Overall, the estimates point to very small effects that are close to zero and mostly statistically insignificant at conventional levels for all six outcomes considered. Hence, the evidence for an effect of the compulsory schooling reforms on health as measured by ICD-coded doctor diagnoses is very weak. First, an explanation for the absence of an effect of the reforms on health might be that the reforms had an effect on schooling duration, but not necessarily on schooling quality. In the context of Germany, the question is what actually happened in schools and what students actually learned in the additional ninth school year. If the teaching content of one school year was spread over two school years, then it is not surprising to find no health benefits. However, according to Leschinsky and Roeder [59], the main objective of introducing the ninth grade in Germany was to expand students’ career and labour market orientation. But it could rather be that that the extra school year improved basic skills, e.g. in numeracy, orthography and language, but not specific skills and knowledge that are required to increase the efficiency in health production or the allocative efficiency of health inputs. Moreover, skills that are important for the labour market might differ from those that are needed for acquiring or processing health information or for producing health more efficiently. Second, an important channel through which education might affect health is an increase in earnings. For Germany, Pischke and van Wachter [51] found that longer schooling does not translate into higher wages, which was confirmed by Kamhöfer and Schmitz [52] with different data. Pischke and van Wachter explain the finding of zero wage returns to compulsory schooling by the fact that the extra school year did not improve skills that are relevant for the labour market because they were already learned earlier in school. In turn, zero returns to education in Germany would rule out one important channel through which education could influence health. However, Cygan-Rehm [53] casts doubts on whether the findings of zero wage returns to education in Germany hold. Third, the lack of a causal impact of education on health might also stem from the fact that returns to education most likely depend on students’ motivation in school. Since the compulsory schooling reforms in West Germany forced some individuals to stay a year longer in school than they actually wanted, motivation might be limited. Accordingly, students who voluntarily attend an additional school year are more likely to benefit from it.

## Discussion and conclusion

The large literature on the causal effect of education on health has found mixed results. This paper aims to add to this strand of literature by carrying out the largest and most comprehensive analysis for Germany to date. Using an ambulatory claims data set that contains ICD-coded doctor diagnoses, we exploit changes in compulsory schooling laws implemented in West Germany in the middle of the twentieth century. Since it depends on month-year of birth whether an individual was affected by the changes in compulsory schooling, the reduced form effect of the reforms on doctor diagnoses is identified within an RD approach.

We find that the reforms have very small impacts on health as measured by doctor-diagnosed conditions in ICD-10 format. In most of the specifications we estimate insignificant effects that are close to zero and often have an unexpected sign. Therefore, our results confirm the findings in Clark and Royer [20], which is the most closely related study to ours in terms of sample size, outcome measures and methodology. The authors estimate very small and mostly insignificant effects of two nationwide reforms in the UK on health measures such as BMI and blood pressure and on self-reported health, which is consistent with our findings for Germany. Moreover, in a recent meta-analysis on the current education-health literature, Xue et al. [46] found that the effect of education on health is close to zero after correcting for positive publication bias. This supports the idea that the results do not depend on the setting and its specific features, but that general education has little effect on health.

Our results have important policy implications. Given the large size of our data set and the wide range of health measures, we think that our study provides important insights into the causal relationship between education and health in the middle to lower parts of the educational distribution. Given that we find small insignificant effects of compulsory schooling on health, our study questions the presence of the sometimes quite large positive effects of education on health that are found in the previous literature and suggests a careful reconsideration of economic models that assume a causal link between education and health. In particular, our results cast doubt on the possibility of developing interventions that efficiently improve both health and education at the same time and on the effectiveness of policies, as suggested by the WHO and OECD, which aim to improve population health through educational interventions. Put differently, educational interventions may not be effective in improving both health and education simultaneously and therefore, policy makers need to implement separate education and health policies, which may lead to trade-offs if one needs to be improved at the expense of the other.

However, our study is subject to some limitations. First, as already discussed above, we construct the treatment indicator using the federal state of current residence to proxy the school attendance state and hence, the treatment might be imprecisely assigned if cross-state mobility in Germany exists. We show, however, that cross-state mobility in Germany is quite low, leading only to small differences in the treatment indicator (see Online Appendix B). This suggests that using the federal state of current residence as a proxy is unlikely to be problematic. Second, the probability of observing individuals in our sample in 2009 would be affected if the changes in compulsory schooling had an effect on mortality. Selective mortality may change the composition of the sample and underestimate the effect of the compulsory schooling laws. So far, there is no study that estimates mortality effects of the German compulsory schooling changes. However, as we concluded earlier that health and mortality effects of compulsory schooling laws are not context-specific and small, we are confident that estimates on mortality effects from other countries also hold true for Germany and that our estimates are not subject to mortality driven sample selection bias. Third, the claims data only include individuals with a least one insurance claim in 2009 and thus, we cannot tell anything about health effects for individuals who did not go to the doctor in 2009. Fourth, diagnoses may be over-reported due to financial incentives. In this context, it has been found that there is a large discrepancy between documented ICD-10 codes and health problems for which the patient has actually been treated [74], which casts doubt on the quality of coding. Moreover, it has been shown that there are regional differences in coding quality [75]. However, this is only problematic for our analyses if coding errors vary systematically at the reform cut-off. This would imply that diagnoses are over-reported to a greater extent among the cohorts that were born before the cut-off and thus affected by the compulsory schooling changes, which seems highly unlikely. Furthermore, regional differences are captured by state fixed effects and state-specific cohort trends in our analyses. Finally, the timing of the compulsory schooling reforms in the 1940s–1960s means that affected individuals were 50–70 years old in 2009. It follows that we do not learn much about the effect of education on health for younger people.

Although we do not find an effect of the compulsory schooling reforms on several health measures, it does not mean that education generally has no effect. The examined compulsory schooling reforms were implemented a long time ago and therefore, we cannot tell anything about health effects of more recent education reforms such as the so-called G8 reform in Germany that exposed students to increased learning intensity [76]. Future research should proceed examining the causal effect of education on health, health behaviours and mortality. It is important to learn about the health effects of interventions at higher levels of education exploiting different sources of exogenous variation as instruments and using samples that are large enough to obtain precise estimates. Since there is only little evidence on the effects of education on more objective measures of health, the particular focus should be on these type of health measures.

## Supplementary Information

Below is the link to the electronic supplementary material.

Supplementary file1 (PDF 305 KB)

## Data Availability

The data that were used in this study are not publicly accessible but available upon request from the Kassenärztliche Bundesvereinigung (KBV).

## Footnotes

1 However, Mazumder [11] shows that her results are not robust to the inclusion of state-specific cohort trends.

2 We thus fulfill all methodological aspects that according to Mazumder [49] are needed for convincing study when exploiting compulsory schooling laws for identification: adopting an RD framework, exploiting month of birth for assigning treatment status and using sufficiently large samples.

3 The reforms have already been found to have zero effects on earnings (see Pischke and van Wachter [51] and Kamhöfer and Schmitz [52]. However, recently, Cygan-Rehm [53] finds that there are positive wage returns to education when reanalysing the data from Pischke and von Wachter. In addition, the reforms have been used to estimate the causal effect of schooling on fertility [54], on intergenerational transmission of education [55], on political behaviour [56] and on pro-immigration attitudes [57].

4 For some of the federal states the introduction dates deviate from the ones reported by Pischke and von Wachter [51], which is the first study considering the German compulsory schooling reform as source of exogenous variation in education. From then on, most studies estimating returns to education in Germany refer to Pischke and von Wachter. A possible explanation for these differences and for the reasons why we rely on the reform dates presented in Table 1 is presented in Online Appendix A.

5 Since the claims data do not contain education, we cannot obtain IV estimates. We have complemented our analysis using the German Socio-Economic Panel (SOEP) to estimate the first stage effect of the reforms on years of schooling and find that the reforms led to an average increase of about 0.5–0.6 years in school (see Online Appendix B, Table 4). However, here we report only reduced form effects of the reforms on health rather than two-sample instrumental variable estimates [61] as we find that the compulsory schooling changes have basically zero effects on these outcomes. Thus, the IV estimates would also be zero.

6 There is a growing literature on the optimal bandwidth choice for an RD estimator [63]. However, we think it is preferable to show the robustness of findings to alternative specifications based on five different bandwidths instead of figuring out the “optimal” bandwidth which is itself subject to random influences.

7 On the federal level, the KBV coordinates the activities of 17 Local Associations of Statutory Health Insurance Physicians (Kassenärztliche Vereinigungen, KVs) in Germany. All office-based physicians and psychotherapists are mandatory members of one of the 17 KVs, depending on their region of work [64]. Physician fees for ambulatory health care services provided to statutorily insured patients are not paid by the patients themselves making advanced payments but directly by the statutory health insurance through the KVs, which are responsible for distributing the fees to the physicians. For this purpose a physician sends an invoice to the statutory health insurance, which contains information on the patient (including year and month of birth as well as place of residence), the ICD-coded diagnoses and the medical serviced performed. A copy of this is forwarded to the KBV [65].

8 It should be mentioned that the numbers reported in Table 3 could be affected by coding errors as physicians may over-report diagnoses to boost profits. We discuss this limitation of the data in more detail in the last section of this paper.

## Code availability

The R code can be made available by the authors upon reasonable request.
