# Supplementary material for: Does compulsory schooling affect health? Evidence from ambulatory claims data
Source: Eur J Health Econ. 2021 Nov 15;23(6):953–68. doi: 10.1007/s10198-021-01404-y (PMC9304067; doi:10.1007/s10198-021-01404-y)
Supplement: Supplementary file 1 — Supplementary file1 (PDF 305 KB) [file 10198_2021_1404_MOESM1_ESM.pdf]

The European Journal of Health Economics

Does compulsory schooling affect health? Evidence from ambulatory claims data

Tatjana Begerow[1] and Hendrik Jürges[2]

[1]Schumpeter School of Business and Economics, University of Wuppertal, Rainer-Gruenter-Str.21 (FN), 42119 Wuppertal, Germany, e-mail: begerow@uni-wuppertal.de.
[2]Schumpeter School of Business and Economics, University of Wuppertal, Rainer-Gruenter-Str.21 (FN), 42119 Wuppertal, Germany, e-mail: juerges@uni-wuppertal.de.

Appendix A: Institutional background

The German school system

In Germany education policy is regulated on the federal state level, but still the school systems are almost identical across federal states [1]. After voluntary pre-school children enter primary school at the age of six and attend it usually for four years. Then they continue schooling in secondary schools. The German secondary school system is tripartite and consists of basic track (Hauptschule), intermediate track (Realschule) and academic track (Gymnasium) that are taught at separate schools, differ by duration and curriculum and lead to different leaving certificates. The basic track is the lowest level of secondary school that provides general basic education and leads to a leaving certificate after eight or nine years, respectively. Students in the intermediate track obtain more extensive general education and graduate after ten years. After finishing basic track or intermediate track school, students usually continue their education starting an apprenticeship or a school-based vocational training. The

academic track is the most demanding and academic-orientated track leading to an university-entrance diploma (Abitur) after grade 13.[1] A lower-level qualification - the technical school degree (Fachhochschulreife) - which allows students to attend a polytechnic (Fachhochschule) can be obtained after finishing grade 12 [3]. In addition, there is a fourth type of secondary school, the so-called comprehensive school (Gesamtschule). This type of secondary school combines all three secondary school tracks at one and the same school. All secondary school leaving certificates can be obtained. However, comprehensive schools do not exist in all states and are rather unimportant with only about 10 percent of all children in Germany attending it [4]. Since the first comprehensive schools were only introduced between 1973 and 1982 on a trial basis [5], this type of school is also relatively new and therefore not relevant for our study as we restrict our analysis to individuals born between 1930 and 1959, who finished secondary school in the late 1970s at the latest.

The allocation to one of the secondary school tracks after primary school depends to a great extent on the student's grades in primary school and thus on student's ability. On that basis, the primary school - usually the class teacher - gives a recommendation for the secondary school type he or she thinks the child should attend after grade four. In ten out of 16 federal states this recommendation is binding, in the remaining six states the final decision is taken by the parents [4, 6]. Since Germany tracks students very early at the age of ten, when information about the student's learning potential is likely to be incomplete, there is a high risk of misallocating students to tracks. In general, there are possibilities for correcting initial track decisions at a later point, when more information about student's ability is available. In principle, switching tracks within secondary schooling is possible at any grade, but in practice this happens rarely. Dustmann et al. [6] show that only about 2 percent of students switch between tracks throughout secondary school. As a consequence, after allocating students into one of the secondary school tracks they generally stay in the chosen track until they complete it. However, up and downgrading of students between tracks at later stages of the educational career is very common. Dustmann et al. provide evidence that there is a substantial movement from the basic and intermediate to the academic track after graduating from the tracks. Moreover, there is a large amount of downgrading in the form of not enrolling in university after graduation from the academic track. Moreover, the authors find that due to this possibility of up and downgrading the assignment to a particular secondary school track at the end of the primary school has only little effect on the highest degree and long-term labour market outcomes.

---

[1] Starting in 2001, a school reform was introduced in most federal states that decreased the length of academic track schooling from 13 to 12 years [2]. As we consider only individuals that attended academic track schools in the 1940s to 1970s this reform is not relevant for our analysis. Thus, we assume 13 years of schooling in the academic track when constructing the schooling variable.

Compulsory schooling reforms: Differences in introduction dates

For some of the federal states our reform introduction dates differ from the ones reported in other studies that use the same German compulsory schooling reforms. Most of these studies refer to Pischke and van Wachter [3], who were the first to exploit the German reforms in order to estimate returns to education. For some federal states there is only a minor difference in the reported dates, for others, e.g. for Saarland and Schleswig-Holstein, the difference is quite large (see Table 1). For Saarland, Pischke and van Wachter [3] report the introduction of the reform in 1964, while we state that it was already in 1958. According to them the compulsory ninth grade was introduced in 1956 in Schleswig-Holstein, but we report that it was already implemented in 1947.

Table 1 Reform introduction dates compared to Pischke and van Wachter [3] and Cygan-Rehm [31]

	Our paper	Pischke and von Wachter (2008)	Cygan-Rehm (2018)
Hamburg	April 1946	1949	1946
Schleswig-Holstein	April 1947	1956	1947
Saarland	April 1958	1964	1958
Bremen	April 1959	1958	1959
Lower Saxony	April 1962	1962	1962
Hesse	April 1966	1967	1967
North Rhine-Westphalia	April 1966	1967	1967
Rhineland-Palatinate	April 1967	1967	1967
Baden-Wuerttemberg	April 1967	1967	1967
Bavaria	August 1969	1969	1969

Notes: The table shows the reform implementation dates used in this paper compared to the dates reported in Pischke and van Wachter [3] and Cygan-Rehm [31] exploiting the same compulsory schooling reforms in West Germany.

One reason for these differences may be that in some federal states it was possible for municipalities to have a voluntary ninth grade before the overall implementation was stipulated by law. Our implementation dates refer to the date when the compulsory ninth grade was implemented in the whole state. Pischke and van Wachter [3] do not provide information, which references they use. We took the information from contemporaneous literature on the German school system by Leschinsky and Roeder [7] and Backhaus [8] and from respective federal state laws [9–16]. Moreover, we reviewed data from the Federal Statistical Office of Germany on the ninth grade attendance in the basic track between 1957 and 1973 [17–30]. Taken together, these references suggest that the implementation dates reported by Pischke and van Wachter [3] are sometimes incorrect. Recent work by Cygan-Rehm [31, 32] questions the reform dates in Pischke and van Wachter [3] as well. Cygan-Rehm also refers to Leschinsky and Roeder [7] and data from the Federal Statistical Office of Germany and reports reform dates that match ours (see Table 1). Two

exceptions are Hesse and North Rhine-Westphalia with a difference of one year each.

Based on data of the Federal Statistical Office of Germany, we plot the number of basic track students in the ninth grade in the school year t as a fraction of the number of basic track students attending the eighth grade in the previous school year t-1 (see Figure 1). Looking at Saarland, for example, we observe that the reform became effective in 1958 as the ninth grade attendance jumps from 0 percent in 1957 to 99 percent in 1958. This matches exactly the timing reported by Leschinsky and Roeder [7] and Backhaus [8] and clearly contradicts the date reported by Pischke and van Wachter [3]. Unfortunately, we do not have early enough data for Schleswig-Holstein to be sure that our reported date is correct. According to Leschinsky and Roeder and Backhaus, the reform introduction was already in 1947, but data is only available from 1957 onwards. However, we are quite confident that the date that was reported by Pischke and van Wachter has to be wrong because the compulsory ninth grade was already stipulated in the state law of Schleswig-Holstein in 1947 [33]. For the remaining federal states our reported reform implementation dates also seem to fit with the data as there are jumps in the ninth grade attendance exactly when the additional school year was fully implemented.

Fig. 1 Number of basic track ninth grade students as a fraction of the number of basic track eighth grade students in the previous school year

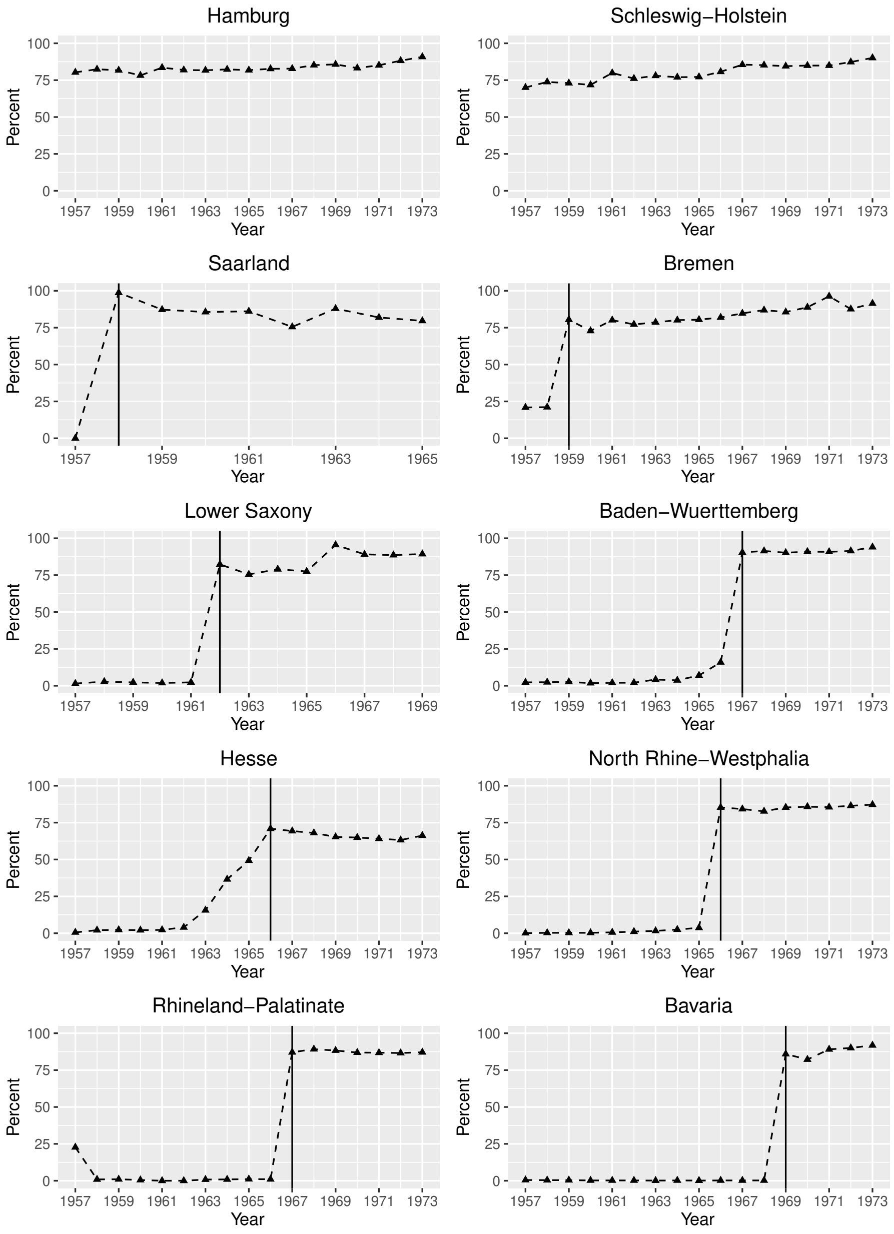

Notes: The figure plots the number of basic track students in the ninth grade in the school year t as a fraction of the number of basic track students attending the eighth grade in the previous school year t-1 for each West German federal state. The vertical line denotes the year of implementation of the reforms. Calculations are based on absolute numbers of basic track students in the eighth and ninth grade from 1956 to 1973. Sources: Federal Statistical Office (1964) and respective volumes of the following years [17–30].

Appendix B: Further analyses using SOEP

Since the KBV claims data do not contain information on years of schooling we are not able to estimate the first stage effect of the compulsory schooling reforms on years of schooling with this data set. However, we complemented our analysis by using the Socio-Economic Panel (SOEP)[2] to estimate a first stage and to test whether measurement error in the treatment indicator due to regional mobility is a potential source of bias for the estimates of the reduced form effect.

The SOEP data

The SOEP is a large representative longitudinal survey of private households in Germany that is conducted annually since 1984 and interviews around 30,000 respondents in nearly 11,000 households every year [34]. For our analysis we pool the waves 1992 to 2018 to obtain a sufficiently large sample. To avoid that some individuals enter the sample more the once, we use for each individual only the observation from 2009 or the observation closest to 2009. Taking observations from 2009 ensures the comparability between the SOEP data and the KBV claims data. Furthermore, we impose the same sample restrictions as on the KBV data. In particular, we consider only German inhabitants of the birth cohorts 1930 to 1959 who are statutorily insured and live in the ten West German federal states excluding Berlin. Furthermore, we exclude individuals that graduated in former East German states or abroad. Moreover, we restrict our sample to individuals with valid information on the federal state of residence, federal state of last school attendance and self-reported health status[3]. For further analyses we consider cohorts born five years before and after each compulsory schooling change.

The SOEP contains only information on the highest secondary school degree. Following Pischke and van Wachter [3], we combine information on an individual's highest secondary school degree and the school years usually required for this degree to construct the schooling variable. Consequently, 13 years are assigned to academic track graduates, if they have university entrance certificate (Abitur) and twelve years, if they have an advanced technical college certificate (Fachhochschulreife). For intermediate track graduates the standard duration of ten years of schooling is taken. For individuals with basic track degree and drop-outs with no school leaving certificate information on the federal state of residence, month and year of birth and the timing of the reform is used to determine whether an individual should have graduated after eight or nine years from the basic track.

Table 2 shows descriptive statistics for the final SOEP sample. The sample consists of 45 percent men, which mirrors almost exactly the share of men in

[2]Socio-Economic Panel (SOEP), data for years 1984-2018, version 35, doi:10.5684/soep-core.v35.

[3]Since 1992 SOEP contains information on self-reported health. We recode the original five-category variable into a dichotomous variable, which is equal to one if individuals report to be in bad or poor health and zero otherwise.

the KVB sample. On average, individuals have a mean age of 57 (compared to 58 in the KBV claims data).[4] Individuals have on average 9.7 years of schooling with women having 0.3 years less than men. The mean probability of self-rated poor or bad health is about the same size for men and women: 23 percent of men and 21 percent of women report to be in poor or bad health. The probability of having an academic school degree is almost twice as large for men. Contrarily, much more women than men have an intermediate school degree. The share of basic school graduates is the same size for men and women: 53 percent of women report to have a basic school degree.[5]

Table 2 Descriptive statistics (SOEP sample)

	(1) Full sample	(2) Men	(3) Women
**Demographics**			
Age	56.5 (5.0)	56.5 (4.9)	56.5 (5.1)
Year of birth	1,950.6 (5.2)	1950.6 (4.9)	1950.6 (5.4)
Male (%)	46.2		
**Health status**			
Self-reported poor or bad health (%)	21.7	23.0	20.7
**Education**			
Years of schooling	9.7 (1.7)	9.9 (1.8)	9.6 (1.5)
Basic track (%)	53.4	53.4	53.4
Intermediate track (%)	26.7	20.4	32.1
Academic track (%)	19.9	26.2	14.6
**Survey information**			
Survey year	2007.7 (2.3)	2007.5 (2.4)	2007.8 (2.2)
N	1,274	588	686

Notes: The table reports mean values of all variables as well as standard deviations of all continuous variables in parentheses for the pooled sample of men and women (column 1) and for the sub-sample of men (column 2) and women (column 3). Source: German Socio-Economic Panel (SOEP).

Potential measurement error in the treatment indicator?

In the KBV claims data only the federal state of residence in 2009 is available, although we would need information on the federal state of last school attendance to ensure a precise assignment of the treatment. By using the state of residence as a proxy, we implicitly assume that individuals attended school in the federal state where they lived in 2009 and thus, the treatment might be

[4] As already mentioned above, we pool several waves of the SOEP and keep the observation from 2009 or closest to 2009 to make the sample comparable to the KBV sample. The survey year in our final SOEP sample is 2008, on average, and thus explains the difference in mean age between the SOEP and KBV samples of about one year.

[5] The probability of having a basic school degree as well as the average number of school years are almost unchanged when we consider the federal state of school attendance instead of the federal state of residence.

imprecisely assigned if cross-state mobility in Germany exists. This measurement error in the treatment indicator would then downward bias our estimates of the treatment effect.

SOEP started collecting information on the federal state of last school attendance in 2001. Using this data, we find that using the state of current residence as a proxy for the school attendance state is unlikely to be problematic. The cross table of the treatment indicators based on the federal state of residence and school attendance in Table 3 shows that cross-state mobility in Germany is quite low, leading only to small differences in the treatment indicator. The table demonstrates that about 95 percent of the people that are coded as treated when the federal state of last school attendance instrument is used are also coded as treated when the instrument is based on the federal state of residence. Moreover, about 96 percent of the non-treated in case of the school attendance instrument are also not treated when the instrument is based on the state of residence.

Table 3 Testing for measurement error in the treatment indicator

	**Federal state of residence**	
**Federal state of last school attendance**	treat=0	treat=1
treat=0	95.9%	4.1%
treat=1	5.1%	94.9%

Notes: The cross-table shows the treatment assignment probability based on the federal state of last school attendance compared to the assignment probability when the the federal state of residence is used to construct the treatment indicator. The table demonstrates that cross-state mobility in Germany is quite low, leading only to small differences in the treatment indicator. Source: German Socio-Economic Panel (SOEP).

We also test whether measurement error in the treatment indicator attenuates IV estimates of the effect of eduction on self-reported health (see Table 4). Therefore, we first estimate the first stage using the instrument based on the federal state of residence and find that the reforms led to an average increase of about 0.56-0.58 years in school (Panel A). The first stage regression results in Panel B show that the assignment of the instrument based on the federal state of school attendance generates slightly smaller first stage coefficients of about 0.52-0.54. Note that the F statistic for the excluded instrument is rather small in both Panels compared to other studies (e.g. Pischke and van Wachter [3] and Kemptner et al. [35]), which can be explained by small sample sizes. Moreover, we find almost unchanged reduced form coefficients when using the federal state of last school attendance instead of the federal state of residence to construct the instrument. Overall, this leads to a very small upward bias in the IV estimates that are based on information on the state of residence. Since the reduced form and IV point estimates are almost identical, we are

quite confident that it is not a major problem to proxy the state of school attendance with the state of residence.

Table 4 IV estimates of the effect of years of schooling on self-reported health

	(1) Local linear	(2) Global linear
**Panel A: Federal state of residence**		
IV parameter	−0.095	−0.090
	(0.088)	(0.085)
First stage parameter	0.559***	0.576***
	(0.186)	(0.184)
Reduced form parameter	−0.053	−0.052
	(0.051)	(0.051)
Observations	1,274	1,274
F-statistic	9.028	9.797
**Panel B: Federal state of last school attendance**		
IV parameter	−0.098	−0.102
	(0.094)	(0.091)
First stage parameter	0.519***	0.544***
	(0.178)	(0.179)
Reduced form parameter	−0.051	−0.056
	(0.048)	(0.048)
Observations	1,274	1,274
F-statistic	8.463	9.262

Notes: The table shows the results from IV regressions of self-reported poor or bad health on years of schooling, based on local linear regressions in column (1) and global linear regressions in column (2), as well as the corresponding reduced form and first stage coefficients. Panel A refers to the instrument based on the federal state of residence, while Panel B relates to the instrument based on the federal state of last school attendance. All regressions are based on the pooled sample of men and women, use a bandwidth of five cohorts around the cut-off point and include fixed effects for year of birth, month of birth and federal state of residence as well as linear state-specific cohort trends, gender and a quadratic age term. Standard errors are clustered on the cohort×state level and presented in parentheses. $^{*}p<0.1$; $^{**}p<0.05$; $^{***}p<0.01$. Source: German Socio-Economic Panel (SOEP).

Appendix C: "Retirement effect"

Fig. 2 Effect of the reforms on depression, musculoskeletal diseases and back pain

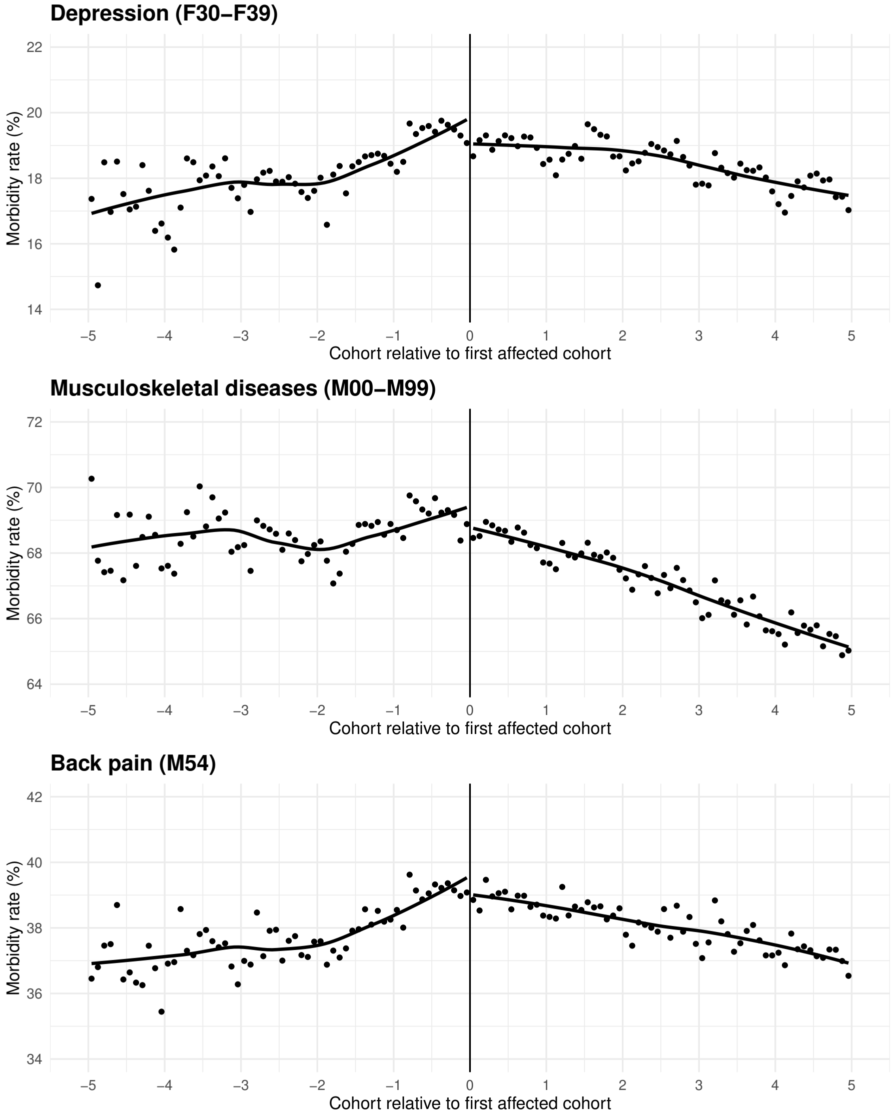

Notes: The figure plots morbidity rates in percent by month-year of birth for five cohorts before and after the first birth cohort affected for the pooled sample of men and women. All data points in the figure present averages by month-year of birth. The vertical line denotes the first birth cohort affected by the law changes. Source: KVB claims data.

References

[1] Dustmann, C.: Parental background, secondary school track choice, and wages. Oxford Economic Papers (2004). https://doi.org/10.1093/oep/gpf048.

[2] Hofmann, S., Mühlenweg, A.: Learning intensity effects in students' mental and physical health – Evidence from a large scale natural experiment in Germany. Economics of Education Review (2018). https://doi.org/10.1016/j.econedurev.2018.10.001.

[3] Pischke, J.S., von Wachter, T.: Zero Returns to Compulsory Schooling in Germany: Evidence and Interpretation. Review of Economics and Statistics (2008). https://doi.org/10.1162/rest.90.3.592.

[4] Jürges, H., Schneider, K.: Why Young Boys Stumble: Early Tracking, Age and Gender Bias in the German School System. German Economic Review (2011). https://doi.org/10.1111/j.1468-0475.2011.00533.x.

[5] Mühlenweg, A.M.: Educational Effects of Alternative Secondary School Tracking Regimes in Germany. Schmollers Jahrbuch (2008). https://doi.org/10.3790/schm.128.3.351.

[6] Dustmann, C., Puhani, P.A., Schönberg, U.: The Long-term Effects of Early Track Choice. The Economic Journal (2017). https://doi.org/10.1111/ecoj.12419.

[7] Leschinsky, A., Roeder, P.M.: Didaktik und Unterricht in der Sekundarstufe I seit 1950 – Entwicklungen der Rahmenbedingungen. In: Max-Planck-Institut für Bildungsforschung: Projektgruppe Bildungsbericht (ed.) Bildung in der Bundesrepublik Deutschland – Daten und Analysen. Bd. 1: Entwicklungen seit 1950, pp. 283-391. Rowohlt, Hamburg (1980).

[8] Backhaus, H.: Das Neunte Schuljahr. Eine Darstellung des Bestandes, der Versuche und der Diskussion. Quelle & Meyer Verlag, Heidelberg (1963).

[9] Gesetzblatt der Freien Hansestadt Bremen. Bekanntmachung des Wortlautes des Gesetzes über das Schulwesen der Freien Hansestadt Bremen vom 25. Mai 1957.

[10] Niedersächsisches Gesetz- und Verordnungsblatt. Gesetz über das öffentliche Schulwesen in Niedersachsen vom 14. September 1954.

[11] Gesetz- und Verordnungsblatt für das Land Nordrhein-Westfalen. Gesetz über die Schulpflicht im Lande Nordrhein-Westfalen (Schulpflichtgesetz-SchPG) vom 14. Juni 1966. https://recht.nrw.de/lmi/owa/br_gv_show_pdf?p_jahr=1966&p_nr=50. Accessed 17 September 2021.

[12] Gesetzblatt für Baden-Württemberg. Gesetz zur Vereinheitlichung und Ordnung des Schulwesens (SchVOG) vom 5. Mai 1964.

[13] Gesetz- und Verordnungsblatt für das Land Rheinland-Pfalz. Erstes Landesgesetz zur Änderung des Schulpflichtgesetzes vom 11. März 1966.

[14] Gesetz- und Verordnungsblatt für das Land Hessen. Hessisches Schulpflichtgesetz vom 17. Mai 1961. http://starweb.hessen.de/cache/GVBL/1961/00013.pdf. Accessed 17 September 2021.

[15] Gesetz- und Verordnungsblatt für das Land Hessen. Gesetz zur Änderung des Hessischen Schulpflichtgesetzes vom 18. November 1965. http://starweb.hessen.de/cache/GVBL/1965/00027.pdf. Accessed 17 September 2021.

[16] Gesetz- und Verordnungsblatt für das Land Bayern. Schulpflichtgesetz (SchPG) vom 15. April 1969. https://www.verkuendung-bayern.de/files/gvbl/1969/06/gvbl-1969-06.pdf. Accessed 17 September 2021.

[17] Federal Statistical Office: Fachserie A, Bevölkerung und Kultur. Reihe 10: Bildungswesen, I. Allgemeinbildende Schulen 1956-1961. Kohlhammer, Stuttgart. https://www.statistischebibliothek.de/mir/receive/DESerie_mods_00004990 (1964). Accessed 17 May 2021.

[18] Federal Statistical Office: Fachserie A, Bevölkerung und Kultur. Reihe 10: Bildungswesen, I. Allgemeinbildende Schulen 1962. Kohlhammer, Stuttgart. https://www.statistischebibliothek.de/mir/servlets/MCRFileNodeServlet/DEHeft_derivate_00037892/FS-A-10-1-1962.pdf (1965). Accessed 17 May 2021.

[19] Federal Statistical Office: Fachserie A, Bevölkerung und Kultur. Reihe 10: Bildungswesen, I. Allgemeinbildende Schulen 1963. Kohlhammer, Stuttgart. https://www.statistischebibliothek.de/mir/servlets/MCRFileNodeServlet/DEHeft_derivate_00037893/FS-A-10-1-1963.pdf (1966). Accessed 17 May 2021.

[20] Federal Statistical Office: Fachserie A, Bevölkerung und Kultur. Reihe 10: Bildungswesen, I. Allgemeinbildende Schulen 1964. Kohlhammer, Stuttgart. https://www.statistischebibliothek.de/mir/servlets/MCRFileNodeServlet/DEHeft_derivate_00037893/FS-A-10-1-1963.pdf (1967). Accessed 17 May 2021.

[21] Federal Statistical Office: Fachserie A, Bevölkerung und Kultur. Reihe 10: Bildungswesen, I. Allgemeinbildende Schulen 1965. Kohlhammer, Stuttgart. https://www.statistischebibliothek.de/mir/servlets/MCRFileNodeServlet/DEHeft_derivate_00037895/FS-A-10-1-1965.pdf (1969). Accessed 17 May 2021.

[22] Federal Statistical Office: Fachserie A, Bevölkerung und Kultur. Reihe 10: Bildungswesen, I. Allgemeinbildende Schulen 1966/67 Kurzschuljahr. Kohlhammer, Stuttgart. https://www.statistischebibliothek.de/mir/servlets/MCRFileNodeServlet/DEHeft_derivate_00037897/FS-A-10-1-1966-67.pdf (1970a). Accessed 17 May 2021.

[23] Federal Statistical Office: Fachserie A, Bevölkerung und Kultur. Reihe 10: Bildungswesen, I. Allgemeinbildende Schulen 1966. Kohlhammer, Stuttgart. https://www.statistischebibliothek.de/mir/servlets/%MCRFileNodeServlet/DEHeft_derivate_00037896/FS-A-10-1-1966.pdf (1970b). Accessed 17 May 2021.

[24] Federal Statistical Office: Fachserie A, Bevölkerung und Kultur. Reihe 10: Bildungswesen, I. Allgemeinbildende Schulen 1967. Kohlhammer, Stuttgart. https://www.statistischebibliothek.de/mir/servlets/MCRFileNodeServlet/DEHeft_derivate_00037898/FS-A-10-1-1967.pdf (1971). Accessed 17 May 2021.

[25] Federal Statistical Office: Fachserie A, Bevölkerung und Kultur. Reihe 10: Bildungswesen, I. Allgemeinbildende Schulen 1968. Kohlhammer, Stuttgart. https://www.statistischebibliothek.de/mir/servlets/MCRFileNodeServlet/DEHeft_derivate_00037899/FS-A-10-1-1968.pdf (1972). Accessed 17 May 2021.

[26] Federal Statistical Office: Fachserie A, Bevölkerung und Kultur. Reihe 10: Bildungswesen, I. Allgemeinbildende Schulen 1969. Kohlhammer, Stuttgart. https://www.statistischebibliothek.de/mir/servlets/MCRFileNodeServlet/DEHeft_derivate_00037900/FS-A-10-1-1969.pdf (1972b). Accessed 17 May 2021.

[27] Federal Statistical Office: Fachserie A, Bevölkerung und Kultur. Reihe 10: Bildungswesen, I. Allgemeinbildende Schulen 1970. Kohlhammer, Stuttgart. https://www.statistischebibliothek.de/mir/servlets/MCRFileNodeServlet/DEHeft_derivate_00037902/FS-A-10-1-1970.pdf (1974). Accessed 17 May 2021.

[28] Federal Statistical Office: Fachserie A, Bevölkerung und Kultur. Reihe 10: Bildungswesen, I. Allgemeinbildende Schulen 1971. Kohlhammer, Stuttgart. https://www.statistischebibliothek.de/mir/servlets/MCRFileNodeServlet/DEHeft_derivate_00038015/FS-A-10-1-1971.pdf (1975). Accessed 17 May 2021.

[29] Federal Statistical Office: Fachserie A, Bevölkerung und Kultur. Reihe 10: Bildungswesen, I. Allgemeinbildende Schulen 1972. Kohlhammer, Stuttgart. https://www.statistischebibliothek.de/mir/servlets/MCRFileNodeServlet/DEHeft_derivate_00037903/FS-A-10-1-1972.pdf (1976a). Accessed 17 May 2021.

[30] Federal Statistical Office: Fachserie A, Bevölkerung und Kultur. Reihe 10: Bildungswesen, I. Allgemeinbildende Schulen 1973. Kohlhammer, Stuttgart. https://www.statistischebibliothek.de/mir/servlets/MCRFileNodeServlet/DEHeft_derivate_00037904/FS-A-10-1-1973.pdf (1976b). Accessed 17 May 2021.

[31] Cygan-Rehm, K.: Is Additional Schooling Worthless? Revising the Zero Returns to Compulsory Schooling in Germany. CESifo Working Paper No. 7191. https://www.ifo.de/DocDL/cesifo1_wp7191.pdf (2018). Accessed 17 May 2021.

[32] Cygan-Rehm, K.: Are there no wage returns to compulsory schooling in Germany? A reassessment. Journal of Applied Economics (2021). https://doi.org/10.1002/jae.2854.

[33] Gesetz- und Verordnungsblatt für Schleswig-Holstein. Gesetz betreffend die Wiedereinführung des 9. Schuljahres vom 11. Februar 1947. http://lissh.lvn.parlanet.de/shlt/lissh-dok/infothek/gvb/1947/XQQGVB475.pdf. Accessed 18 May 2021.

[34] Wagner, G.G., Frick, J.R., Schupp, J.: The German Socio-Economic Panel Study (SOEP): Scope, Evolution and Enhancements. Schmollers Jahrbuch: Zeitschrift für Wirtschafts- und Sozialwissenschaften 127(1), 139-170 (2007).

[35] Kemptner, D., Jürges, H., Reinhold, S.: Changes in compulsory schooling and the causal effect of education on health: Evidence from Germany. Journal of Health Economics (2011). https://doi.org/10.1016/j.jhealeco.2011.01.004.
